# *Vibrio* Species and Cyanobacteria: Understanding Their Association in Local Shrimp Farm Using Canonical Correspondence Analysis (CCA)

**DOI:** 10.1007/s00248-024-02356-5

**Published:** 2024-03-15

**Authors:** Awg Baki Dayang Najwa, Nillian Elexson, Lesen Dalene, Sing Tung Teng

**Affiliations:** Faculty of Resource Science and Technology, University Malaysia Sarawak, 94300 Kota Samarahan, Sarawak Malaysia

**Keywords:** *Vibrio*, *Vibrio cholerae*, *Vibrio parahaemolyticus*, Cyanobacteria, Environmental microbiology, Microbial interactions

## Abstract

In aquatic environments, *Vibrio* and cyanobacteria establish varying relationships influenced by environmental factors. To investigate their association, this study spanned 5 months at a local shrimp farm, covering the shrimp larvae stocking cycle until harvesting. A total of 32 samples were collected from pond A (*n* = 6), pond B (*n* = 6), effluent (*n* = 10), and influent (*n* = 10). *Vibrio* species and cyanobacteria density were observed, and canonical correspondence analysis (CCA) assessed their correlation. CCA revealed a minor correlation (*p* = 0.847, 0.255, 0.288, and 0.304) between *Vibrio* and cyanobacteria in pond A, pond B, effluent, and influent water, respectively. Notably, *Vibrio* showed a stronger correlation with pH (6.14–7.64), while cyanobacteria correlated with pH, salinity (17.4–24 ppt), and temperature (30.8–31.5 °C), with salinity as the most influential factor. This suggests that factors beyond cyanobacteria influence *Vibrio* survival. Future research could explore species-specific relationships, regional dynamics, and multidimensional landscapes to better understand *Vibrio*-cyanobacteria connections. Managing water parameters may prove more efficient in controlling vibriosis in shrimp farms than targeting cyanobacterial populations.

**Supplementary Information:**

The online version contains supplementary material available at 10.1007/s00248-024-02356-5.

## Introduction

Shrimp farming is one of the most significant products for the Malaysian aquaculture industry. Among the Association of Southeast Asian Nations (ASEAN) countries, Malaysia has maintained its competitiveness in exporting fresh shrimp, alongside Thailand [1]. In Sarawak, the state’s Modernization of Agriculture and Regional Development Minister aims to expand shrimp farming with the goal of producing RM 1 billion in exports by 2030 (Sulok [2]). The extensive shrimp farm selected for this study achieved the production of 165 mt of shrimp in 2021 [3], which marks great potential for the goal. As shrimp farming in Malaysia has the potential to be profitable and contribute to the country’s economic expansion, effective disease prevention measures are essential to avoid profit loss from bacterial infections.

The shrimp farming industry regards vibriosis as a significant threat as it can affect the entire life cycle of shrimp, ranging from eggs to brood stock, which may lead to the complete eradication of an entire population in case there is an outbreak [4, 5]. These infections can spread rapidly within shrimp ponds due to the proximity and crowded conditions in aquaculture settings [6]. The *Vibrionecea*e family has captivated scientists due to its adaptability in the aquaculture environment. In the marine ecosystem, their prevalence and pathogenicity in the environment raise concerns, as *Vibrio* is still endemic in certain countries [7]. They were also recognized to interact with other bacterial groups [8] and other aquatic creatures in the context of an aquatic environment [9]. *Vibrio* species possess a curved morphology and exhibit flagellated motility on one end, measuring 0.5–0.8 μm in width and 2–3 μm in length [10]. In clinical microbiology and food safety, *V. cholerae*, *V. parahaemolyticus*, *V. vulnificus*, *V. alginolyticus*, and *V. mimicus* are among the *Vibrio* species that are potentially dangerous [11].

It has been demonstrated through previous research that cyanobacteria play a crucial role in the persistence of *Vibrio* in our environment [12]. These bacteria possess the unique ability to conduct photosynthesis, and they exist in either coccoid unicellular, coccoid multicellular, or filamentous forms [13]. Certain cyanobacterial species coexist in habitats alongside *Vibrio*, providing dissolved organic matter that sustains the *Vibrio* community [14]. These bacteria, classified as opportunistic in nature, exhibit rapid proliferation in specific conditions such as low oxygen levels, insufficient nourishment, and contaminated water [15]. These environmental stressors, resulting from the opportunistic behavior of both cyanobacteria and *Vibrio*, can also adversely impact shrimp. As microorganisms are very capable of fostering symbiotic relationships with each other, plants, and animals, more research is required to better understand them.

Despite the evidence of their association, there have been a number of studies that have demonstrated contradictory results. Some isolated strains of cyanobacteria have been found to exhibit antibacterial properties against pathogenic microorganisms, indicating that they can potentially eradicate *Vibrio* species instead of nurturing them [16]. Another study has found that the relationship between cyanobacteria and *Vibrio* species depends on the growth stage of the cyanobacteria, which indicates that the association is not without specific circumstances [17]. To accommodate the differences in findings, there is a need to conduct research on the interaction between cyanobacteria and *Vibrio* species, particularly in the context of shrimp farms, where favorable conditions for the proliferation of both bacteria genera are naturally present.

Aside from the potential association with cyanobacteria, water parameters such as temperature, salinity, and pH can also influence the growth and abundance of opportunistic *Vibrio* species [18] in shrimp farms. High water temperatures, for example, were proven to provide optimal conditions for the growth of *Vibrio* species [19]. Shrimps are equally sensitive to changes in their environment, and stressors such as suboptimal water quality can weaken their immune systems [20], making them more susceptible to infections from *Vibrio* species. On the contrary, the maintenance of optimum salinity can lead to better survival, weight gain, and protein retention rates in reared shrimp [21].

To find out the correlation between both *Vibrio* species and cyanobacteria with water parameters, a statistical method called canonical correspondence analysis (CCA) was employed. Previous studies have used CCA as a tool to analyze the microbial communities of aquatic environments, and it has been effective in elucidating the correlation between *Vibrio* populations, particulate organic matter, and environmental factors [22]. By implementing CCA as the statistical method, the microbial ecology consisting of the association between both genera and the water parameters was studied.

This study is focused on the aquaculture of shrimp and encompasses several objectives. These include quantifying the abundance of *Vibrio* species and cyanobacteria within a selected shrimp farm, identifying the presence of two prominent *Vibrio* species known to pose risks to both humans and shrimps, and determining the specific genera of cyanobacteria present in that environment, followed by assessing potential *Vibrio*-cyanobacteria correlations with water quality parameters. It is hypothesized that the association between *Vibrio* species and cyanobacteria in shrimp farms is highly dependent on environmental factors such as water temperature, salinity, and pH. Methods such as most probable number (MPN), duplex-polymerase chain reaction (D-PCR), Sedgewick-Rafter cell counting, differential interference contrast (DIC) microscopy using an inverted microscope, and canonical correspondence analysis (CCA) were implemented in this study to achieve the objectives of this study.

## Experimental Procedures

### Water Sampling

**Fig. 1:**
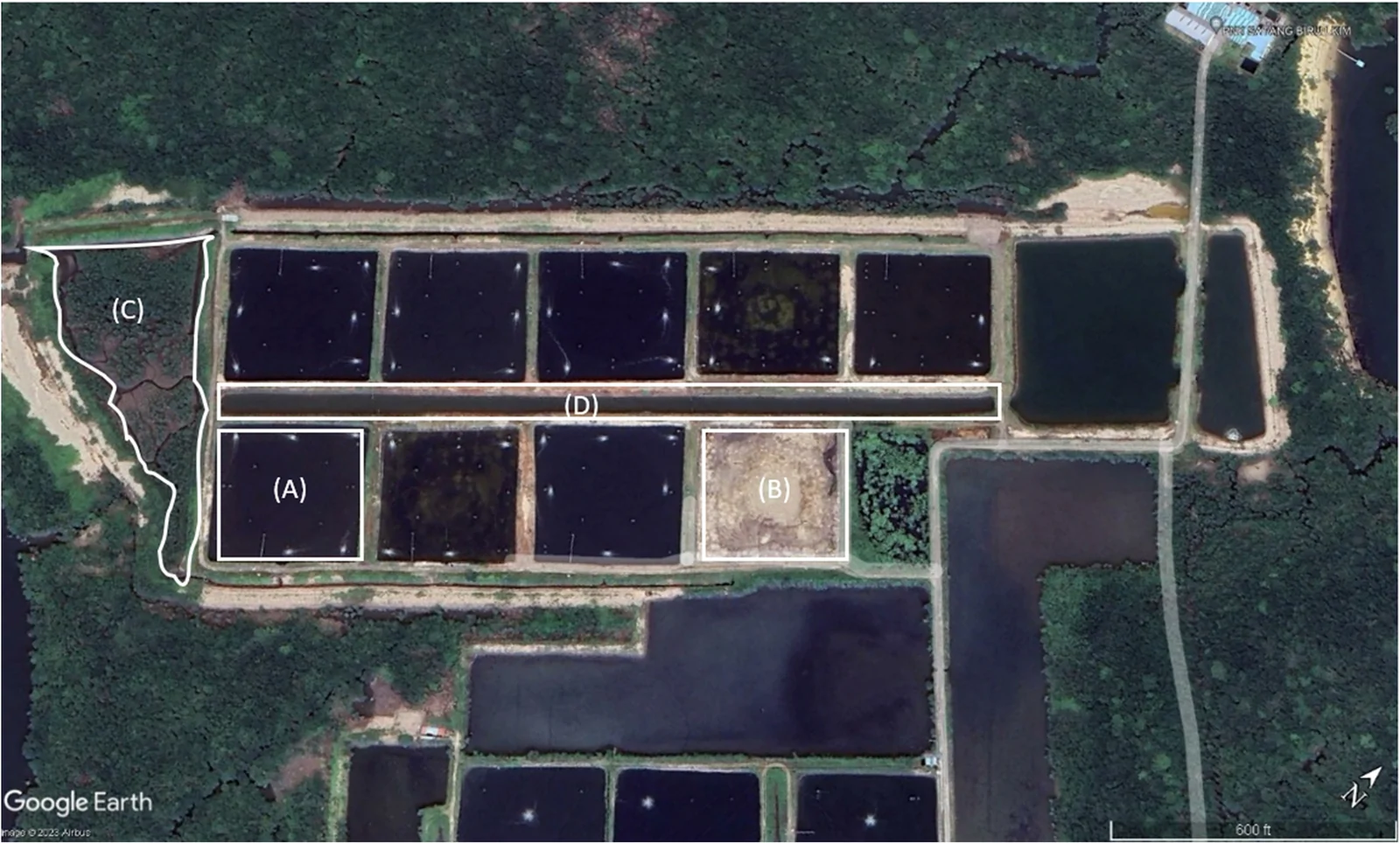
Map of the sampling sites at Persatuan Nelayan Kawasan Satang Biru, Telaga Air, Kuching, Sarawak, Malaysia: (**A**) pond A, (**B**) pond B, (**C**) effluent, and (**D**) influent

Water sampling was commenced biweekly (August–December 2021) in two white-leg shrimp (*Litopenaeus vannamei*) in shrimp ponds (pond A and pond B) and effluent and influent water (Fig. 1) of Persatuan Nelayan Kawasan Satang Biru, Telaga Air, Kuching, Sarawak, Malaysia (1° 40′ 34.7″ N, 110° 12′ 10.8″ E). The sampling campaign was executed within the shrimps’ post-larvae stocking to harvesting time frame. The shrimp-rearing ponds have a surface area of 1 ha, a depth of 1.3 m, and a stocking density of 40 post-larvae (PL)/m^2^. A total of six (*n* = 6) samples from pond A, six (*n* = 6) samples from pond B, ten (*n* = 10) samples from effluent, and ten (*n* = 10) samples from influent were successfully collected, making up thirty-two (*n* = 32) samples. A plankton net was used to sift water from shrimp ponds to collect a concentrated amount of cyanobacteria inside 50-mL Falcon tubes. Environmental parameters such as water temperature, salinity, and pH were also recorded during the sampling activity—Fisherbrand^TM^ accumet^TM^ AP125 Portable pH/Ion/mV/temperature meter kit (Thermo Fischer Scientific, USA) for the temperature and pH and refractometer (STAT, China). The samples were then transported to the laboratory under aseptic conditions within 2 h at room temperature.

### Enrichment and Dilution of Vibrio Species

A total of 1 mL of the water samples were pipetted into 9 mL of Alkaline Peptone Water (APW) (HiMedia, India) to enrich *Vibrio* species in the samples. The cultures were then incubated overnight at 37 °C. Remaining water samples were treated with Lugol’s solution for the cyanobacteria counting step. After enrichment, 1 mL of culture broths was diluted up to 10^−5^. Each dilution was incubated overnight at 37 °C.

### Most Probable Number (MPN)

MPN method from the US Food and Drug Administration’s Bacterial Analytical Manual (BAM) [23] was employed to enumerate the *Vibrio* species present in each water sample. This is done by making serial dilutions of the water samples and inoculating aliquots into broth media to detect bacterial growth. Firstly, 1 mL of the broths from the selected dilutions (10^−2^, 10^−3^, and 10^−4^) was inoculated into triplicates of 10 mL APW. The culture broths were then incubated for 24 h at 37 °C. The tubes that went turbid from each triplicate of MPN tubes from every dilution indicate growth for *Vibrio* species. The number of tubes that went turbid in each triplicate was recorded, and the result was referred to the MPN table [23]. In this study, we used a different approach compared to the traditional MPN table for presenting our results. Instead of using dilutions of 10^−1^, 10^−2^, and 10^-3^ like in a normal MPN table, the present study used dilutions of 10^−2^, 10^−3^, and 10^−4^. This modification allowed us to obtain accurate measurements and evaluate the microbial population more effectively. Therefore, each result for MPN per milliliter was multiplied by 10 to match the table configuration [24]. From each MPN tube that was incubated, a loopful of broth was streaked on the surface of thiosulfate-citrate-bile salts sucrose (TCBS) agar (HiMedia, India) and subsequently incubated overnight at 37 °C for *Vibrio* species confirmation.

### DNA Extraction of Vibrio Species

The method was modified from Peng et al.’s [25] to fit the requirements of this study. Five hundred microliters of the culture broth was pipetted into a 1.5-mL microcentrifuge tube and centrifuged at 10,000 rpm for 5 min. The supernatant was discarded before adding 100 μL of deionized distilled water (ddH_2_O). The mixture was then boiled for 10 min and snap-cooled in ice for 5 min. Lastly, each tube was centrifuged for 10 min at 10,000 rpm. The clear solution is the product of the DNA extract.

### Polymerase Chain Reaction (PCR)

PCR was done by adding exTEN PCR 2X Master Mix (Base Asia), 2 pairs of forward and reverse primers [26] as specified in Table 1, nuclease-free water, and DNA extracts from each sampling. In each PCR reaction, 12.5 μL of exTEN 2X PCR Master Mix (Base Asia) was used as the PCR reagent. The forward primers VP 1155272 F and VC C634002 F, as well as the reverse primers VP 1155272 R and VC C634002 R, were added at a volume of 0.6 μL each, with a concentration of 10 pmol/μL. Nuclease-free water was included at a volume of 1.1 μL. Additionally, 1.0 μL of DNA extract was added to the reaction mixture. The total volume of each PCR reaction was 17 μL.

**Table 1:** Primer pairs and their sources used for the identification of *V. cholerae* and *V. parahaemolyticus*

Target genus or species	Primer name	Protein of target gene	Primer sequences	PCR base pair
*V. parahaemolyticus*	VP 1155272 F	Hypothetical protein VPA1095	5’ - AGCTT ATTGG CGGTT TCTGT CGG – 3’	297
	VP 1155272 R		5’ - CCAA GACCA AGAAA AGCCG TC – 3’	
*V. cholerae*	VC C634002 F	Hypothetical protein VCA0694	5’ - CAAGC TCCGC ATGTC CAGAA GC – 3’	154
	VC C634002 R		5’ - GGGGC GTGAC GCGAA TGATT – 3’	

The amplification of the DNA sequence was done by using a thermal cycler (Eppendorf Mastercycler Gradient) and with an initial denaturation step, performed at 94 °C for 6 min. Following this, denaturation takes place at 94 °C for 30 s. Annealing is conducted at 62 °C for 2 min, repeated for a total of 35 cycles. Subsequently, extension is carried out at 72 °C for 1 min and 50 s. Lastly, a final extension step occurs at 72 °C for 6 min.

### Agarose Gel Electrophoresis (AGE)

The PCR products were loaded into 1.5% agarose gel and were charged with 80 V electric current for 1 h prior to being stained for 45 min with 0.1% ethidium bromide (EtBr). The bands formed were then observed by using a UV transilluminator before being photographed.

### Quantification and Morphological Identification of Cyanobacteria

A total of 1 mL of water samples that were previously preserved with Lugol’s solution were pipetted by using a Pasteur pipette into Sedgewick-Rafter counting chambers exactly filling it while avoiding the formation of air bubbles. The water sample was then given about 5–10 min to settle down before being counted grid by grid using a compound microscope starting from left to right [27]. This process was repeated 3 times for each sample to gather the average count of the result. The number of cells per filament of the cyanobacteria was obtained by multiplying the total filaments counted with the average number of cells of the first 30 filaments [28]. The morphological identification of cyanobacteria was done by using an inverted microscope: Olympus FluoView 300 (Olympus Corporation, Japan). The microscope incorporates the differential interference contrast (DIC) filter in the process that can produce high-quality, pseudo-3D images of cyanobacteria (Scientifica, n.d.). The camera used to capture the images was called Infinity 3 (Teledyne Lumenera, Ottawa, Canada), while the software used to analyze the image was Infinity Analyze (Teledyne Lumenera, Ottawa, Canada).

### Canonical Corresponding Analysis (CCA)

Data regarding the environmental variables obtained during the sampling activity and *Vibrio* and cyanobacteria populations was used to recognize any significant relationship between all the variables. The *p*-values are provided to indicate the statistical significance of *Vibrio* species and cyanobacteria. A *p*-value that is greater than 0.01 is considered not significant, while a *p*-value that is less than or equal to 0.01 indicates a significant relationship between the variables. The analysis was performed with PAST software version 4.03 (Palaeontological Association, Norway) which is freely available on the Internet at the time of writing.

## Results

### Environmental Parameters

Based on Table 2, the pond water pH ranges from 6.14 to 7.64, indicating that they were slightly acidic to neutral. While influent water was neutral, pond B was shown to develop the greatest amount of acidity on average. Minimal changes in water temperature for both ponds, effluent, and influent can be observed as they only slightly fluctuate. The salinity of ponds A and B both generally increased as the sampling process came to a close, contributing to the mean of 24. Evidently, the influent water had the lowest salinity compared to all sampling sites.

**Table 2:** A summary on the selected environmental parameters involved in this study

Sampling sites	Duration of sampling	Total samples	Water pH (mean),	Water temperature (mean), (°C)	Water salinity (mean), (ppt)
Pond A	10 August -19 October 2020	6	± 6.59	± 31.5	± 24
Pond B	5 October – 14 December 2020	6	± 6.14	± 30.8	± 24
Effluent	10 August – 14 December 2020	10	± 6.57	± 30.9	± 23
Influent	10 August – 14 December 2020	10	± 7.64	± 30.9	± 17.4

### Growth Pattern and Identification of Vibrio Species

#### Pond A

**Fig. 2:**
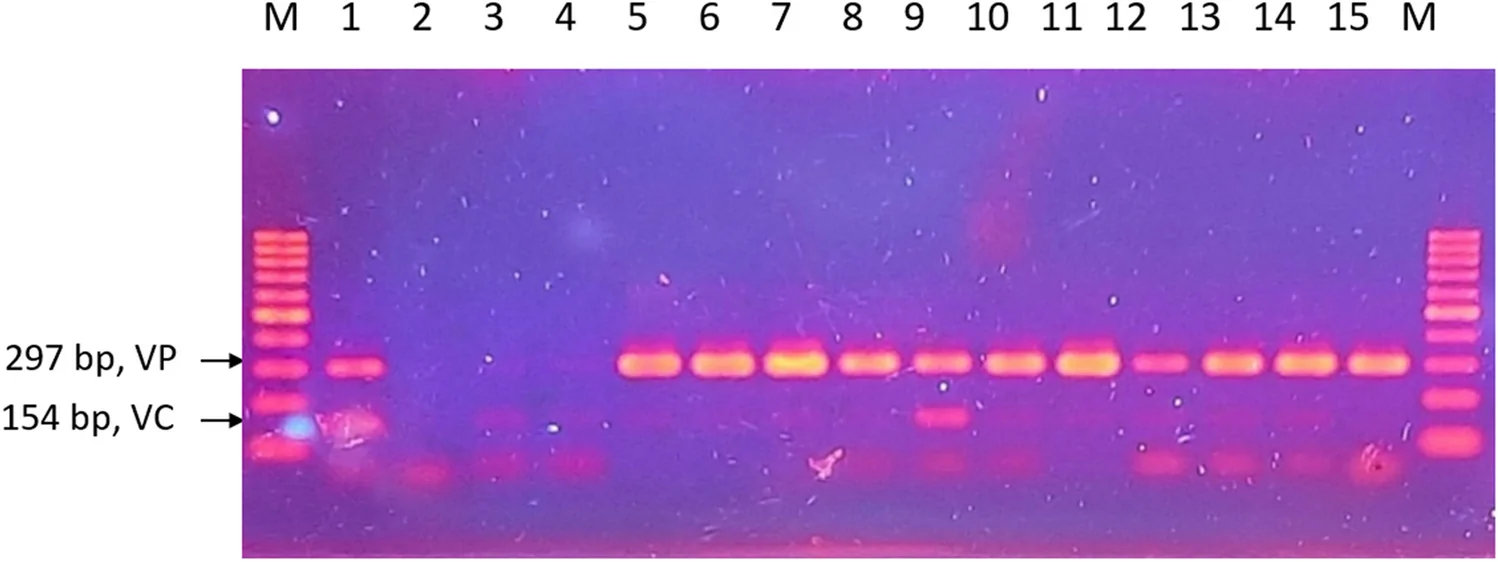
Duplex PCR result for the detection of V. parahaemolyticus and V. cholerae for pond A viewed in 1.5% agarose gel. M, 100 bp DNA ladder; 1, positive control of V. cholerae and V. parahaemolyticus; 2, negative control; 3, sampling 1; 4, sampling 2; 5–7, sampling 3; 8–9, sampling 4; 10–12, sampling 5; 13–15, sampling 6. Positive detection for V. parahaemolyticus is seen in lane 3 to 15, while positive detection for V. cholerae is seen in lane 3 to 14. V. cholerae bands appear to be faint when compared to V. parahaemolyticus

The growth of *Vibrio* showed a notable rise during the second sampling and remained consistent until the conclusion of all samplings (Table 3). *Vibrio* species continued to be abundant until the end of the sampling cycle. In summary, PCR detection of *V. cholerae* and *V. parahaemolyticus* in pond A shows 72% positive detection of *V. cholerae* and 78% of *V. parahaemolyticus* (Fig. 2).

**Table 3:** The growth profile of *Vibrio* species in Pond A

Number of samplings	Sampling site triplicates	MPN/10 mL
Sampling 1	A	>11000
	B	<30
	C	<30
Sampling 2	A	<30
	B	>11000
	C	<30
Sampling 3	A	>11000
	B	>11000
	C	>11000
Sampling 4	A	>11000
	B	>11000
	C	>11000
Sampling 5	A	>11000
	B	>11000
	C	>11000
Sampling 6	A	>11000
	B	>11000
	C	>11000

#### Pond B

**Fig. 3:**
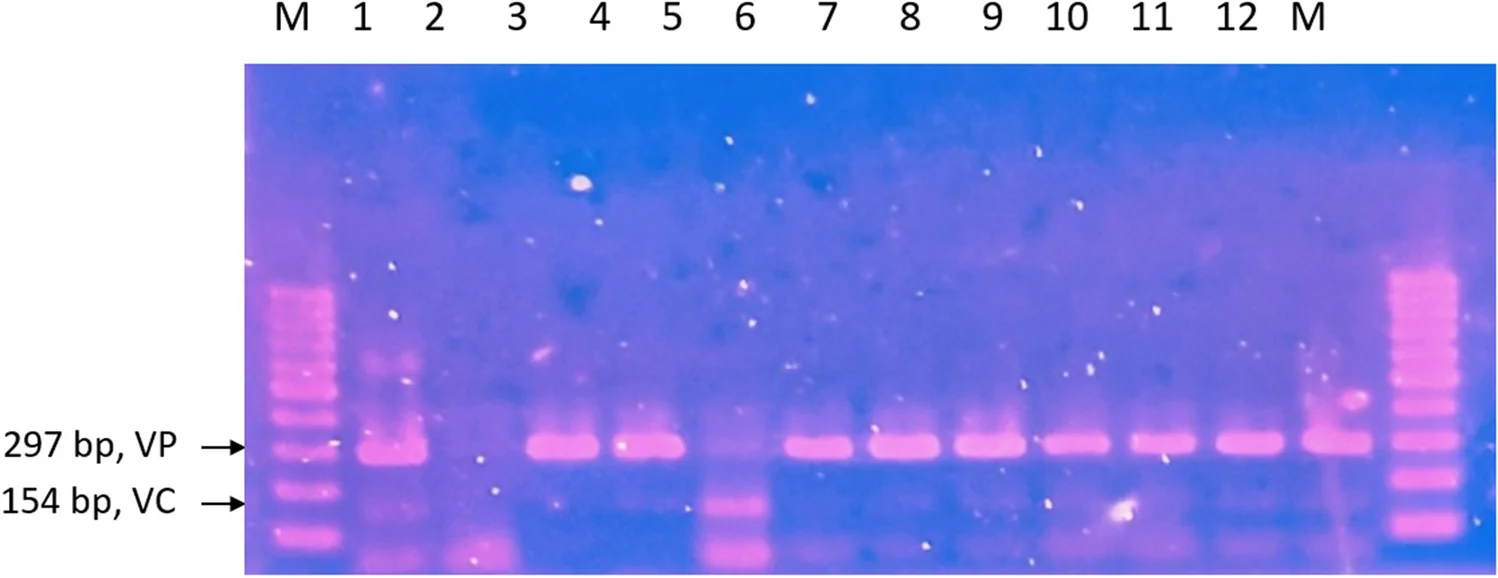
Duplex PCR result for the detection of V. parahaemolyticus and V. cholerae for pond B viewed in 1.5% agarose gel. M, 100 bp DNA ladder; 1, positive control of V. cholerae and V. parahaemolyticus; 2, negative control; 3 and 4, sampling 1; 5, sampling 2; 6, sampling 3; 7 and 8, sampling 4; 9 and 10, sampling 5; 11 and 12, sampling 6. All samples show positive detection of V. parahaemolyticus, while the positive detection of V. cholerae is shown in lanes 4, 5, 7, 8, 9, 10, 11, and 12. V. cholerae bands appear to be faint when compared to V. parahaemolyticus with the exception of lane 5

The *Vibrio* species demonstrated rapid growth, reaching peak levels during each sampling period (Table 4). According to the findings obtained through the duplex-PCR technique, *V. cholerae* and *V. parahaemolyticus* were detected with a prevalence of 61% and 100%, respectively (Fig. 3).

**Table 4:** The growth profile of *Vibrio* species in Pond B

Number of samplings	Sampling site triplicates	MPN/10 mL
Sampling 1	A	>11000
	B	>11000
	C	>11000
Sampling 2	A	>11000
	B	>11000
	C	>11000
Sampling 3	A	>11000
	B	>11000
	C	>11000
Sampling 4	A	>11000
	B	>11000
	C	>11000
Sampling 5	A	>11000
	B	>11000
	C	>11000
Sampling 6	A	>11000
	B	>11000
	C	>11000

#### Effluent

**Fig. 4:**
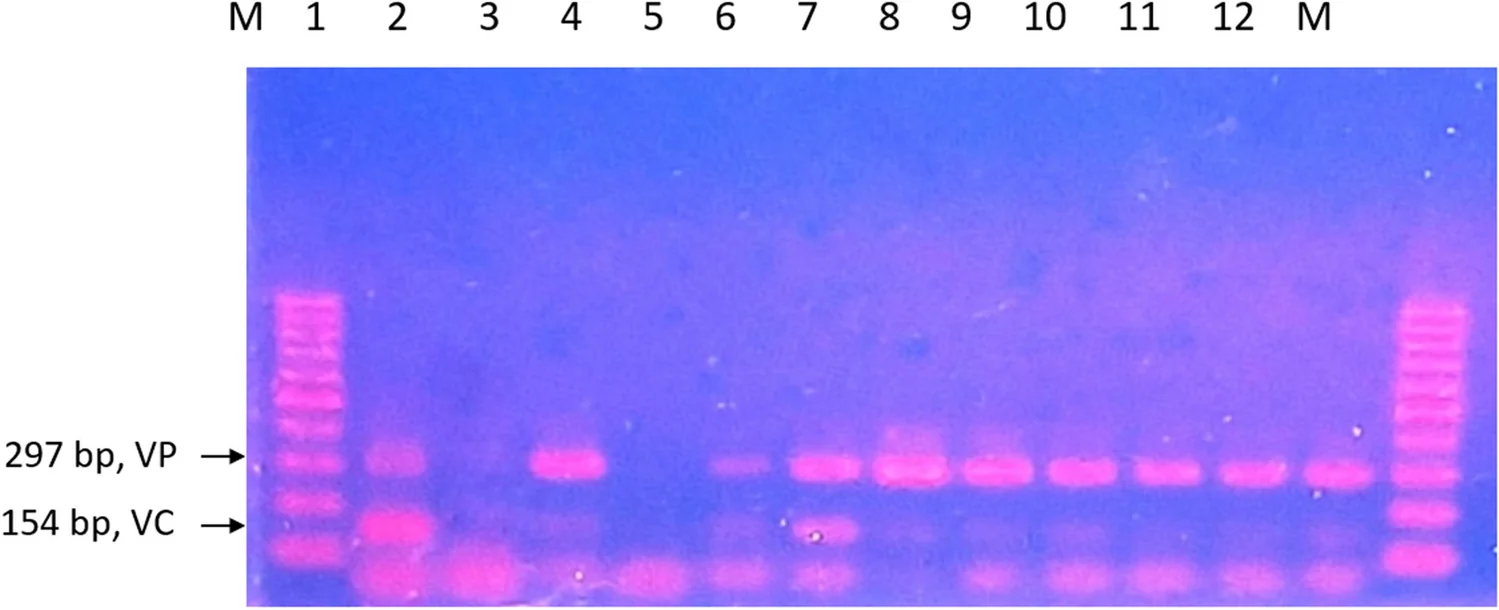
Duplex PCR result for the detection of V. parahaemolyticus and V. cholerae for effluent water viewed in 1.5% agarose gel. M, 100 bp DNA ladder; 1, positive control of V. cholerae and V. parahaemolyticus; 2, negative control; 3 and 4, sampling 2; 5, sampling 3; 6, sampling 4; 7, sampling 5; 8, sampling 6; 9, sampling 7; 10, sampling 8; 11, sampling 9; 12, sampling 10. Positive detection of V. parahaemolyticus can be seen in lanes 3, 5, 6, 7, 8, 9, 10, 11, and 12, same with V. cholerae. V. cholerae bands appear to be faint when compared to V. parahaemolyticus with the exception of lane 6

The initial effluent water sampling demonstrated minimal *Vibrio* proliferation, while the following samples indicated a rapid escalation in bacterial growth that remained consistent until the completion of the sampling period (Table 5). The detection of *V. cholerae* and *V. parahaemolyticus* gathered 90% of both species’ prevalence and can be observed in Fig. 4.

**Table 5:** The growth profile of *Vibrio* species in effluent water

Number of samplings	MPN/10 mL
Sampling 1	<30
Sampling 2	>11000
Sampling 3	>11000
Sampling 4	>11000
Sampling 5	>11000
Sampling 6	>11000
Sampling 7	>11000
Sampling 8	>11000
Sampling 9	>11000
Sampling 10	>11000

#### Influent

**Fig. 5:**
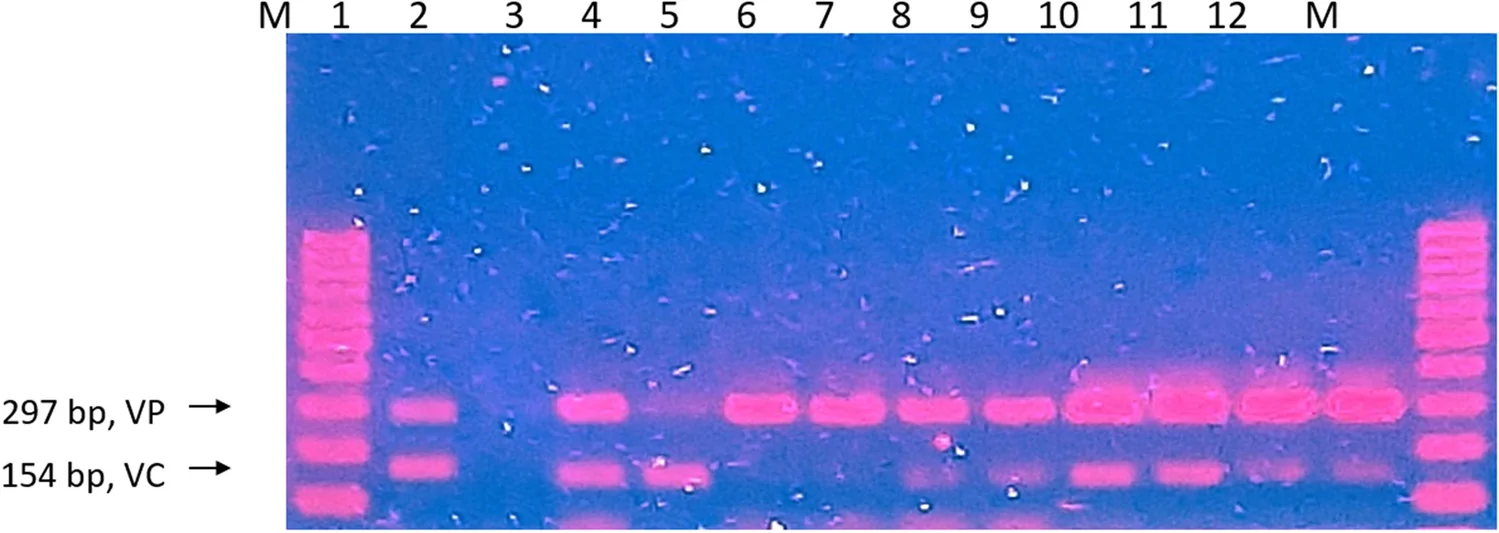
Duplex PCR result for the detection of V. parahaemolyticus and V. cholerae for influent water viewed in 1.5% agarose gel. M, 100 bp DNA ladder; 1, positive control of V. cholerae and V. parahaemolyticus; 2, negative control; 3, sampling 1; 4, sampling 2; 5, sampling 3; 6, sampling 4; 7, sampling 5; 8, sampling 6; 9, sampling 7; 10, sampling 8; 11, sampling 9; 12, sampling 10. Positive detection of V. parahaemolyticus can be seen on lanes 3 to 12, while positive detection of V. cholerae can be seen on lanes 3, 4, 7, 8, 9, 10, 11, and 12. V. cholerae bands are faint in comparison to V. parahaemolyticus with the exception of lane 4

*Vibrio* growth can be seen off to a gradual increase during earlier sampling before the number persistently soared from the third sampling until the end (Table 6). As observed in Fig. 5, the duplex-PCR result of *V. cholerae* and *V. parahaemolyticus* showed successful detection of both species with 70% of *V. cholerae* and 90% of *V. parahaemolyticus* prevalence.

**Table 6:** The growth profile of *Vibrio* species in influent water

Number of samplings	MPN/10 mL
Sampling 1	150
Sampling 2	350
Sampling 3	>11000
Sampling 4	>11000
Sampling 5	>11000
Sampling 6	>11000
Sampling 7	>11000
Sampling 8	>11000
Sampling 9	>11000
Sampling 10	>11000

### Growth Pattern and Identification of Cyanobacteria

**Fig. 6:**
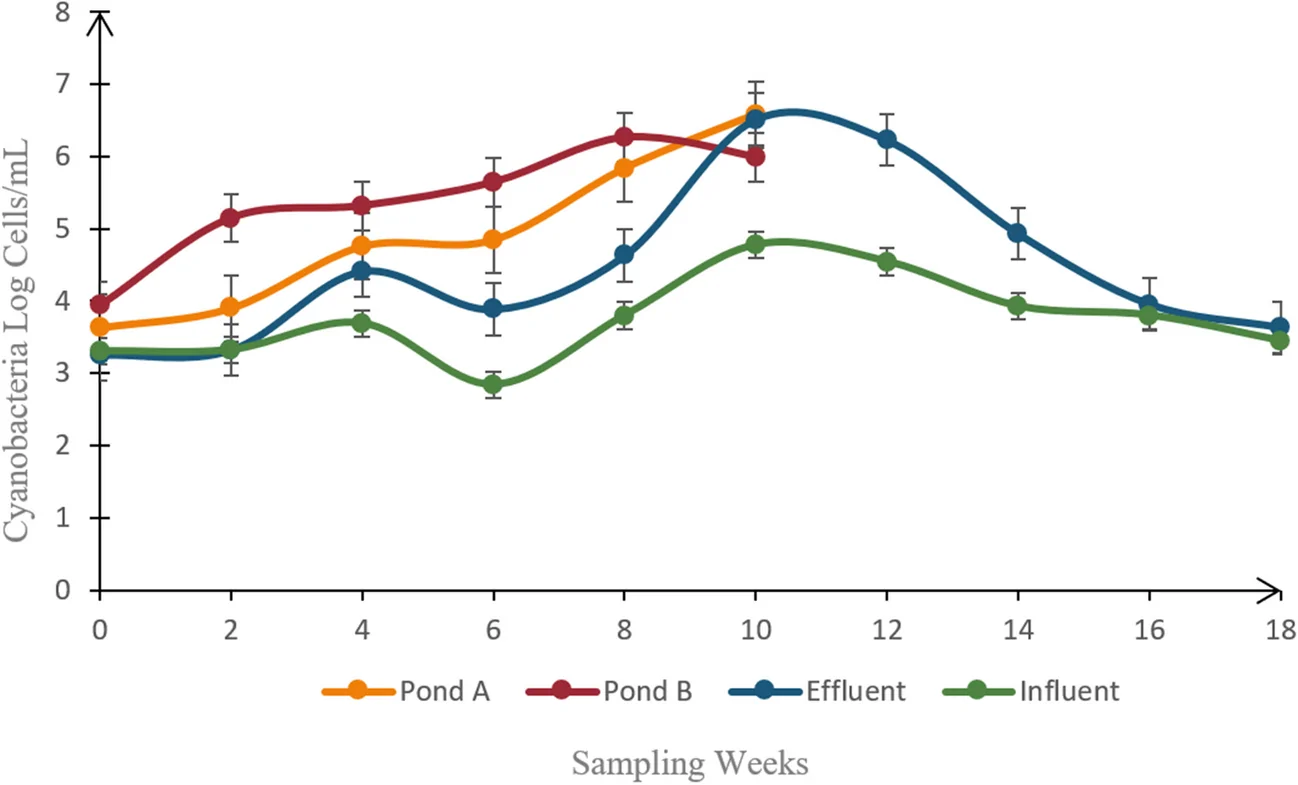
The cyanobacteria cell growth pattern in the shrimp farm

Figure 6 shows the growth pattern of cyanobacteria in every sampling site, across their sampling period. Pond A’s cyanobacterial growth curve gradually increased during the sample weeks. The 4th and 6th weeks, however, show a stagnant growth development. As for pond B, up until the 8th week, cyanobacterial growth indicated an overall increase. After that, there was a slight dip in the growth curve during week 10. The overall growth pattern for effluent displayed two peaks, one during the 4th week and the other in the 10th. However, it was clear that the peak of the 10th week was higher than the peak of the 4th week. The growth curve started to decline after the 10th week. The growth of cyanobacteria in the influent water can be observed to fluctuate, thus forming two peaks, which occurred during the 4th and 10th week. In comparison to the peak from the 4th week, the peak from the 10th week appeared to be higher. There was also a noticeable decline in growth during the 6th week, which appeared to be at the lowest point throughout the entire sampling period of this site.

A total of five members of the cyanobacteria genera were found residing in the shrimp farm water, namely, *Chroococcus*, *Pseudanabaena*, *Phormidium*, *Oscillatoria*, and *Lyngbya*. Based on cell counting with a Sedgewick-Rafter cell and morphological examination with an inverted microscope, it was evident that early samplings accumulate a greater diversity of cyanobacteria when compared to the conclusion of the sampling period, where *Pseudanabaena* genus can be seen dominating. The morphological assessment of the cyanobacterial species can be referred to in Table 7.

**Table 7:**
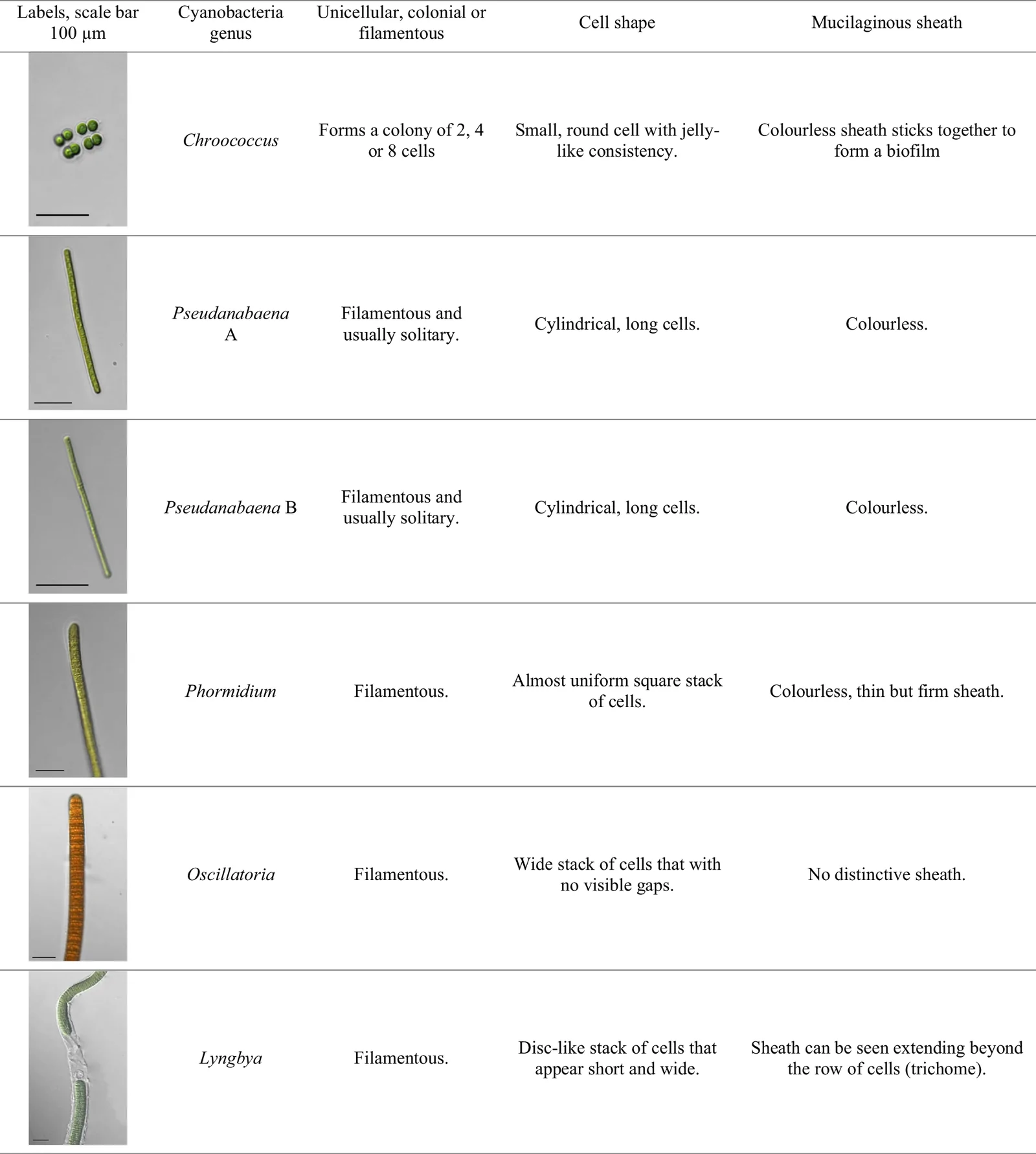
The morphological assessment of observed cyanobacteria in the shrimp farm water

### Canonical Correspondence Analysis (CCA)

**Fig. 7:**
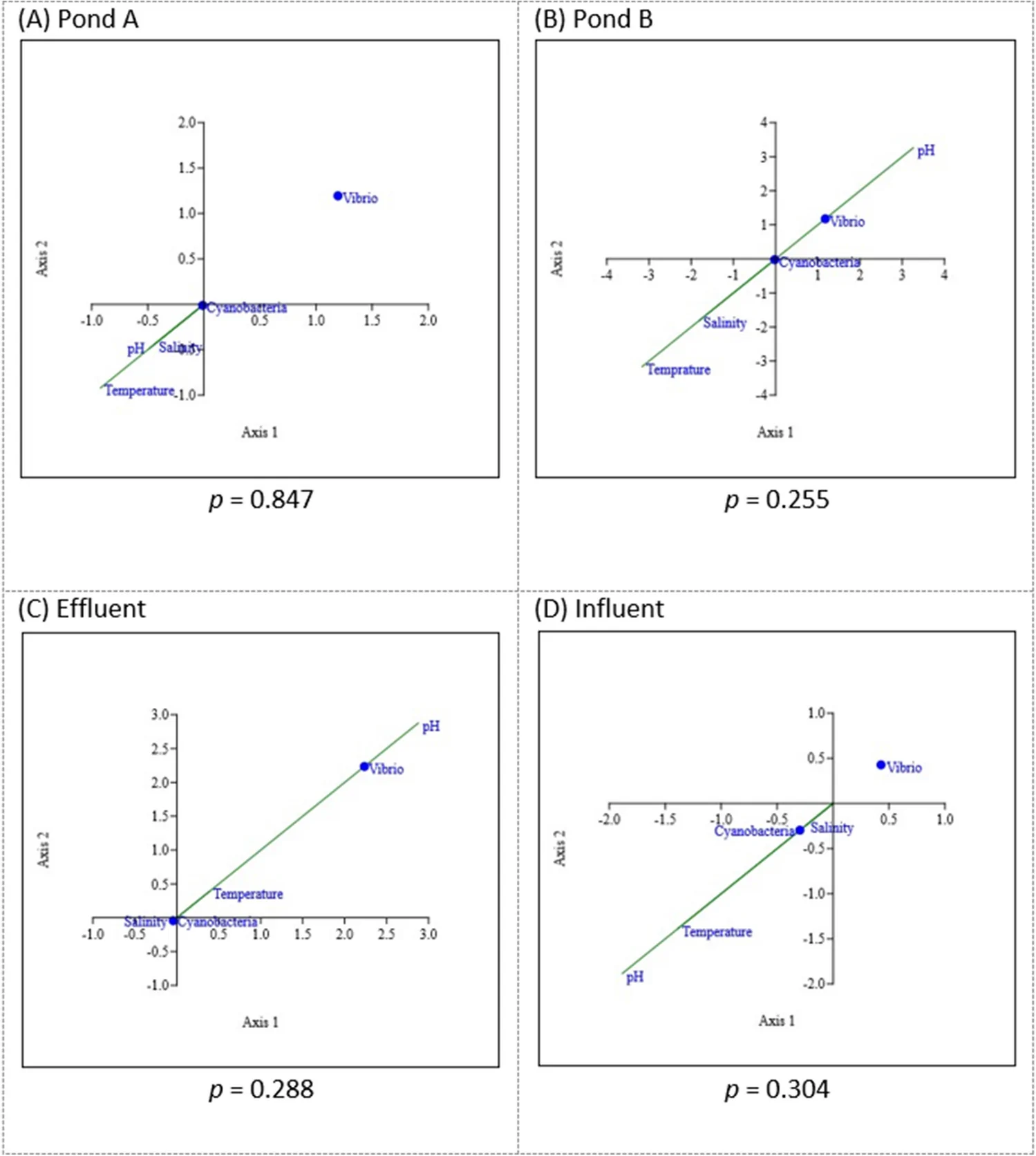
**A**–**D** The products of canonical correspondence analysis as calculated using PAST software

The association between *Vibrio* species and cyanobacteria in pond A, pond B, effluent, and influent water is shown in Fig. 7. Canonical correspondence analysis (CCA) was used to calculate the *p*-value for the correlation between both bacteria species. The water parameters were included to determine the distribution of *Vibrio* species and cyanobacteria according to abiotic factors. All *p*-values are insignificant (*p* > 0.01).

Figure 7A shows that the proliferation of cyanobacteria in pond A is predominantly associated with pH, salinity, and temperature as opposed to *Vibrio* species. The CCA for pond B (Fig. 7B) shows that *Vibrio* growth gravitates toward pH, while cyanobacteria growth gravitates toward salinity and temperature, although a minimal correlation between both bacteria can be seen. In effluent water (Fig. 7C), a connection can be seen between *Vibrio* and cyanobacteria, yet the growth of *Vibrio* is still primarily associated with pH levels rather than cyanobacteria, temperature, and salinity. Influent water shows no correlation between *Vibrio* species and cyanobacteria; however, cyanobacteria correlate with all environmental parameters (Fig. 7D).

In summary, both genera have a stronger relationship with environmental factors than they do with one another. The link between both genera is proven to be inconsistent with every sampling site and is minor. Cyanobacteria can be seen to prefer salinity at all sample locations, while *Vibrio* species were observed to prefer pH in pond B and effluent water. Compared to *Vibrio* species, cyanobacteria were seen to be more closely related to all water parameters.

## Discussion

This study aimed to investigate the associations between *Vibrio* species and cyanobacteria in different sampling sites in a single shrimp farm, namely, pond A, pond B, effluent water, and influent water. CCA was used to examine the correlation between these bacteria species with respect to various abiotic factors such as pH, salinity, and temperature. The results showed that there is a stronger relationship between both genera and environmental parameters than between themselves. Cyanobacteria were found to prefer salinity at all sample locations, while *Vibrio* species were observed to have a preference for pH levels in pond B and effluent water. Overall, these findings underscore the complexity of the relationship between *Vibrio* species and cyanobacteria.

Water quality parameters act as a measure of the health and well-being of shrimp culture [29]. Maintaining ideal environmental conditions can ensure shrimps’ resilience to bacterial infections, to which they are particularly vulnerable [30]. In the sampling location where this study was conducted, pH, salinity, and temperature were monitored daily by the farmers. The mean pH for all the sampling sites was recorded to be along the line of 6.14 to 7.64. A moderate increase in the acidity of the shrimp farm water was observed as the sampling progressed toward the end. This might indicate the accumulation of decaying organic matter in shrimp farm waters [31]. This observation can be supported by the low pH displayed by pond A, pond B, and effluent that are in contact with shrimps but not from influent where the water comes into the shrimp farm from an outside source. Despite being slightly acidic, the level of pH remained in the normal range of 6.5 to 9.0 [32].

In previous studies, it has been observed that shrimps are highly sensitive to even slight variations in water temperature, which can have profound effects on their size [33]. Optimal shrimp yields have been reported at water temperatures ranging from 23.5 to 25.5 °C or between 30 and 31.5 °C [34]. Furthermore, the recommended range for maintaining ideal conditions during shrimp rearing is generally recognized as being between 28 and 32 °C [32]. In line with these findings, consistent water temperatures averaging from 30.8 to 31.5 °C were recorded in this study without significant fluctuations present. The water temperatures in the shrimp farm were within the optimal range for shrimp rearing, promoting favorable growth and yield. This result can be attributed to the consistent temperature characteristics of the coastal regions in Malaysia.

As for salinity, the optimum growing performance for shrimp was discovered between 20 and 30 ppt [35]. Likewise, the recorded salinity of the ponds (17.4 to 24 ppt) was well kept within the ideal range in all sampling sites. Shrimps can tolerate changes in salinity level as long as proper acclimation was done before stocking the post-larvae [36]. It is clear from the environmental parameters’ data that daily monitoring in shrimp farms is essential in ensuring the best possible water quality for the shrimps’ growth.

In addition to the parameters explored in this study, it is essential to acknowledge the role of another factor, the dissolved oxygen (DO) level, which also holds importance in ensuring the well-being of a shrimp farm ecosystem. The shrimp farmer involved in the research reported that the recorded range of DO on the farm was 5–8 mg/L, surpassing the optimum levels recommended by Robertson [37], which suggested 4 or 5 mg/L. The heightened DO levels observed in the shrimp farm can be attributed to the installation of an aeration system designed to ensure adequate DO levels [38]. The presence of phytoplankton, particularly through photosynthesis, also serves as the primary source of DO in water [39]. Additionally, factors that can have an effect on DO concentrations in water include temperature and salinity. When water temperature and salinity increase, oxygen solubility decreases [40, 41]. Recognizing the potential linkage between DO levels and the complex interactions among *Vibrio* species, cyanobacteria, and overall shrimp farm health, future research should address the study’s limitations by incorporating comprehensive dissolved oxygen data.

The interspecies relationship between the two genera has been proven to be an extremely complex discussion. In this research, a comparison was made between the growth of *Vibrio* bacteria and cyanobacteria in relation to different water parameters. Through the use of CCA, it was determined that pH, salinity, and temperature all play a role in affecting the growth of cyanobacteria; however, salinity had a more significant impact on their proliferation than other factors. This has also been proven by previous research [42]. Salinity can affect cyanobacteria in many ways such as their cell growth and their rate of photosynthesis [43]. Cyanobacteria’s tolerance toward salt might be due to the fact that they contain a vast regulatory mechanism that allows them to acclimatize in various salt concentrations [44]. Cyanobacteria’s high tolerance toward salt has been proven beneficial in the past for treating saline soil to improve rice crops [45]. In pond B and effluent water, *Vibrio* species were found to favor pH. However, other ponds (pond A and influent) indicate no major effect of pH toward the growth of *Vibrio* species. The variation in the result might indicate the optimum pH where they grow best [46].

Our finding is consistent with previous studies where a minimal interspecies correlation between *Vibrio* species and cyanobacteria was documented [47–49]. The differences in growth patterns between *Vibrio* species and cyanobacteria account for this phenomenon. When compared to the overall *Vibrio* species which population tends to be perpetually high, the growth curve of cyanobacteria displayed a clearer consistency. They have a structure resembling the lag phase, exponential or log phase, stationary phase, and death phase like normal bacterial growth. This is possible due to the naturally slow growth rate of cyanobacteria due to their ability to carry on complex mechanisms (such as the synthetization of amino acid and utilization of carbon dioxide for photosynthesis) causing the cells to prioritize cell function over reproduction [50]. In contrast to cyanobacteria, heterotrophic bacteria like *Vibrio* species use less energy to proliferate because they consume rather than produce their food, leading to their quicker and more rapid development profile [50]. This observation is also in line with a previous investigation conducted in the Pacific Northwest region of the USA in 2015, which reported minimal influence on *Vibrio* concentration due to the low abundance of cyanobacteria gathered [49]. Other aforementioned studies demonstrate that the interaction between the two genera is not a result of random events but rather influenced by various factors. The specific species involved [48], seasonal temperatures [47], and regional characteristics [51] play significant roles in determining this interspecies relationship. However, this finding should not completely rule out the compatibility of *Vibrio* proliferation with cyanobacterial blooms as 43–64% of the cyanobacterial bloom samples exhibited an association of viable but nonculturable forms of *V. cholerae* [52].

Throughout the sampling period, *Vibrio* species can be seen to either kick-start their growth slowly followed by a drastic increase in growth or maintain a constant high growth pattern above 1000 UFC/mL which is the maximum range of acceptable *Vibrio* population in healthy shrimp farm water [32]. *V. cholerae* was detected at rates of 72%, 61%, 90%, and 70% in pond A, pond B, effluent, and influent (respectively), while *V. parahaemolyticus* was identified at levels of 78%, 100%, 90%, and 90% using duplex-PCR methodology (Table 8). The detection of both *Vibrio* species is expected since they are naturally present in aquatic habitat ([53, 54]); however, the high concentrations are unforeseen due to the well-monitored water parameters. High concentrations of *Vibrio* in water are commonly viewed as a rather undesirable attribute as they can cause outbreaks of shrimp diseases [55]. Despite that, it is important to consider that not all *Vibrio* species in shrimp farms are pathogenic ([53, 54]); therefore, further testing for toxin genes was done to analyze the potential of the *Vibrio* species in causing infection. From the result, pronounced variation in the *V. cholerae* and *V. parahaemolyticus* prevalence can be explained by the halophilic profile of *V. parahaemolyticus* [7] which thrives in saline water better than *V. cholerae*. *V. parahaemolyticus* can also sustain a greater range of environmental differences such as pH and temperature in coastal water than *V. cholerae* [56]. Since the sampling was done in a coastal region, it is expected that *V. parahaemolyticus* population exceeds that of *V. cholerae* which explains the brighter bands displayed in duplex PCR.

**Table 8:** The summary of the PCR detection of *V. cholerae* and *V. parahaemolyticus* in Pond A, Pond B, effluent and influent

*Vibrio* species	Number and percentage of positive detection			
	Pond A	Pond B	Effluent	Influent
*V. cholerae*	13/18 (72%)	11/18 (61%)	9/10 (90%)	7/10 (70%)
*V. parahaemolyticus*	14/18 (78%)	18/18 (100%)	9/10 (90%)	9/10 (90%)

Cyanobacteria may be very well distinguished from other bacteria based on the characteristics of their cell dimensions, shape, color, type of branching, sheath characteristics, and cell contents (summarized in Komárek and Anagnostidis [57–60]). They possess easily visible features that are easy to recognize, even at lower magnifications with light microscopy [61, 62]. Staining is rarely needed in cyanobacterial microscopy due to the presence of chloroplasts in their cells as natural blue-green pigments [63]. Some also contain red/brownish color due to a variety of pigments, like carotenoids and phycobiliproteins [63]. In the present study, it is apparent that the *Pseudanabaena* genus dominated the shrimp farm water as we approach the end of the sampling, significantly reducing the diversity of cyanobacteria. Aside from the reason that the *Pseudanabaena* genus can commonly be found inhabiting planktonic water, they also are highly tolerant toward disturbance and low light when compared to other cyanobacteria genera [64]. *Chroococcus*, *Phormidium*, *Oscillatoria*, and *Lyngbya* were among the other species that were discovered during prior samplings. In a nutshell, the study of cyanobacteria diversity in shrimp farms can lead to a better knowledge of their ecological roles, risk factors, and interactions with the shrimp farming system.

It can be concluded that environmental considerations such as pH, temperature, and salinity are more important factors in determining the growth of *Vibrio* species and cyanobacteria than their relationship with each other. It is suggested that reducing exposure to environmental stressors may be a more effective way to limit *Vibrio* and cyanobacterial growth than focusing on controlling one bacterium over another. In addition to water parameters, the monitoring of the dissolved oxygen level [65] and the population density of *Vibrio* species is suggested for the early prevention of shrimp diseases [55]. Additionally, several limitations of our study are the focus on a specific shrimp farming location which may limit the generalizability of our findings to other geographic regions or aquaculture systems, aside from the deficiency of DO level data. Further research is required to determine the precise mechanisms by which these bacteria interact with their environment and to develop more targeted interventions for managing them in aquaculture systems. A conclusive study involving a multidimensional landscape with a wider scope to multiple locations can link fragmentary knowledge regarding the relationship of both genera.

## Conclusion

This study investigated the relationship between *Vibrio* species and cyanobacteria in a local shrimp farm. The results indicate that while there is a minor association between *Vibrio* species and cyanobacteria, other factors appear to have a stronger influence on the survival of *Vibrio* species. Specifically, pH levels were found to be more strongly correlated with the presence of *Vibrio* populations than cyanobacteria. Cyanobacteria, on the other hand, showed associations with factors such as pH, salinity, and temperature, with salinity playing a particularly influential role. Since other factors affecting the persistence of *Vibrio* species in shrimp farms may be present still, further studies should also explore other factors such as species-specific relationships, regional dynamics, and other multidimensional landscapes that may be affecting the persistence of *Vibrio* species in a shrimp farm. It is suggested that controlling water parameters can be more efficient in controlling vibriosis in shrimp farms compared to controlling cyanobacterial populations.

Dayang Najwa, A. B., Elexson, N., Dalene, L., & Teng, S. T. (2024). Vibrio Species and Cyanobacteria: Understanding their Association in Local Shrimp Farm using Canonical Correspondence Analysis (CCA). Microbial Ecology. 87(1). 10.1007/s00248-024-02356-5

## Supplementary information

ESM 1 (DOCX 28 kb)

## Data Availability

All data generated or analyzed in this study are included in this published article.
