# Supplementary material for: Vibrio Species and Cyanobacteria: Understanding Their Association in Local Shrimp Farm Using Canonical Correspondence Analysis (CCA)
Source: Microb Ecol. 2024 Mar 15;87(1):51. doi: 10.1007/s00248-024-02356-5 (PMC10943157; doi:10.1007/s00248-024-02356-5)
Supplement: Supplementary file 1 — (DOCX 28 kb) [file 248_2024_2356_MOESM1_ESM.docx]

**Supplementary Information**

The abundance of *Vibrio* species (MPN/10mL) in Pond A**.**

| Number of samplings | Sampling site triplicates | Number of positive tubes | | | MPN/10mL | 95% Confidence Range |
| --- | --- | --- | --- | --- | --- | --- |
|  |  | 0.01 | 0.001 | 0.0001 |  |  |
| Sampling 1 | A | 3 | 3 | 3 | >11000 | 4200-40000 |
|  | B | 0 | 0 | 0 | <30 | 0-95 |
|  | C | 0 | 0 | 0 | <30 | 0-95 |
| Sampling 2 | A | 0 | 0 | 0 | <30 | 0-95 |
|  | B | 3 | 3 | 3 | >11000 | 4200-40000 |
|  | C | 0 | 0 | 0 | <30 | 0-95 |
| Sampling 3 | A | 3 | 3 | 3 | >11000 | 4200-40000 |
|  | B | 3 | 3 | 3 | >11000 | 4200-40000 |
|  | C | 3 | 3 | 3 | >11000 | 4200-40000 |
| Sampling 4 | A | 3 | 3 | 3 | >11000 | 4200-40000 |
|  | B | 3 | 3 | 3 | >11000 | 4200-40000 |
|  | C | 3 | 3 | 3 | >11000 | 4200-40000 |
| Sampling 5 | A | 3 | 3 | 3 | >11000 | 4200-40000 |
|  | B | 3 | 3 | 3 | >11000 | 4200-40000 |
|  | C | 3 | 3 | 3 | >11000 | 4200-40000 |
| Sampling 6 | A | 3 | 3 | 3 | >11000 | 4200-40000 |
|  | B | 3 | 3 | 3 | >11000 | 4200-40000 |
|  | C | 3 | 3 | 3 | >11000 | 4200-40000 |

The abundance of *Vibrio* species (MPN/10mL) in Pond B.

| Number of samplings | Sampling site triplicates | Number of positive tubes | | | MPN/10mL | 95% Confidence Range |
| --- | --- | --- | --- | --- | --- | --- |
|  |  | 0.01 | 0.001 | 0.0001 |  |  |
| Sampling 1 | A | 3 | 3 | 3 | >11000 | 4200-40000 |
|  | B | 3 | 3 | 3 | >11000 | 4200-40000 |
|  | C | 3 | 3 | 3 | >11000 | 4200-40000 |
| Sampling 2 | A | 3 | 3 | 3 | >11000 | 4200-40000 |
|  | B | 3 | 3 | 3 | >11000 | 4200-40000 |
|  | C | 3 | 3 | 3 | >11000 | 4200-40000 |
| Sampling 3 | A | 3 | 3 | 3 | >11000 | 4200-40000 |
|  | B | 3 | 3 | 3 | >11000 | 4200-40000 |
|  | C | 3 | 3 | 3 | >11000 | 4200-40000 |
| Sampling 4 | A | 3 | 3 | 3 | >11000 | 4200-40000 |
|  | B | 3 | 3 | 3 | >11000 | 4200-40000 |
|  | C | 3 | 3 | 3 | >11000 | 4200-40000 |
| Sampling 5 | A | 3 | 3 | 3 | >11000 | 4200-40000 |
|  | B | 3 | 3 | 3 | >11000 | 4200-40000 |
|  | C | 3 | 3 | 3 | >11000 | 4200-40000 |
| Sampling 6 | A | 3 | 3 | 3 | >11000 | 4200-40000 |
|  | B | 3 | 3 | 3 | >11000 | 4200-40000 |
|  | C | 3 | 3 | 3 | >11000 | 4200-40000 |

The abundance of *Vibrio* species (MPN/10mL) in effluent water.

| Number of samplings | Number of positive tubes | | | MPN/10mL | 95% Confident Range |
| --- | --- | --- | --- | --- | --- |
|  | 0.01 | 0.001 | 0.0001 |  |  |
| Sampling 1 | 0 | 0 | 0 | <30 | 0-95 |
| Sampling 2 | 3 | 3 | 3 | >11000 | 4200-40000 |
| Sampling 3 | 3 | 3 | 3 | >11000 | 4200-40000 |
| Sampling 4 | 3 | 3 | 3 | >11000 | 4200-40000 |
| Sampling 5 | 3 | 3 | 3 | >11000 | 4200-40000 |
| Sampling 6 | 3 | 3 | 3 | >11000 | 4200-40000 |
| Sampling 7 | 3 | 3 | 3 | >11000 | 4200-40000 |
| Sampling 8 | 3 | 3 | 3 | >11000 | 4200-40000 |
| Sampling 9 | 3 | 3 | 3 | >11000 | 4200-40000 |
| Sampling 10 | 3 | 3 | 3 | >11000 | 4200-40000 |

The abundance of *Vibrio* species (MPN/10mL) in influent water.

| Number of samplings | Number of positive tubes | | | MPN/10mL | 95% Confident Range |
| --- | --- | --- | --- | --- | --- |
|  | 0.01 | 0.001 | 0.0001 |  |  |
| Sampling 1 | 2 | 1 | 0 | 150 | 37-420 |
| Sampling 2 | 2 | 2 | 2 | 350 | 87-940 |
| Sampling 3 | 3 | 3 | 3 | >11000 | 4200-40000 |
| Sampling 4 | 3 | 3 | 3 | >11000 | 4200-40000 |
| Sampling 5 | 3 | 3 | 3 | >11000 | 4200-40000 |
| Sampling 6 | 3 | 3 | 3 | >11000 | 4200-40000 |
| Sampling 7 | 3 | 3 | 3 | >11000 | 4200-40000 |
| Sampling 8 | 3 | 3 | 3 | >11000 | 4200-40000 |
| Sampling 9 | 3 | 3 | 3 | >11000 | 4200-40000 |
| Sampling 10 | 3 | 3 | 3 | >11000 | 4200-40000 |
